# A Validation of Supervised Deep Learning for Gait Analysis in the Cat

**DOI:** 10.3389/fninf.2021.712623

**Published:** 2021-08-19

**Authors:** Charly G. Lecomte, Johannie Audet, Jonathan Harnie, Alain Frigon

**Affiliations:** Department of Pharmacology-Physiology, Faculty of Medicine and Health Sciences, Centre de Recherche du CHUS, Université de Sherbrooke, Sherbrooke, QC, Canada

**Keywords:** gait analysis, deep learning, markerless pose estimation, cat, kinematics

## Abstract

Gait analysis in cats and other animals is generally performed with custom-made or commercially developed software to track reflective markers placed on bony landmarks. This often involves costly motion tracking systems. However, deep learning, and in particular DeepLabCut^TM^ (DLC), allows motion tracking without requiring placing reflective markers or an expensive system. The purpose of this study was to validate the accuracy of DLC for gait analysis in the adult cat by comparing results obtained with DLC and a custom-made software (Expresso) that has been used in several cat studies. Four intact adult cats performed tied-belt (both belts at same speed) and split-belt (belts operating at different speeds) locomotion at different speeds and left-right speed differences on a split-belt treadmill. We calculated several kinematic variables, such as step/stride lengths and joint angles from the estimates made by the two software and assessed the agreement between the two measurements using intraclass correlation coefficient or Lin’s concordance correlation coefficient as well as Pearson’s correlation coefficients. The results showed that DLC is at least as precise as Expresso with good to excellent agreement for all variables. Indeed, all 12 variables showed an agreement above 0.75, considered good, while nine showed an agreement above 0.9, considered excellent. Therefore, deep learning, specifically DLC, is valid for measuring kinematic variables during locomotion in cats, without requiring reflective markers and using a relatively low-cost system.

## Introduction

Millennia ago, our ancestors illustrated animals in movement (e.g., hunting or fleeing), as depicted in the cave of Lascaux, France. Around 350 B.C., Aristotle, a Greek philosopher, wrote *De motu animalium*, latin for Movement of animals (Nussbaum, 1976), considered the first textbook dealing with the analysis of movement and locomotion. Later on, in the 1870s, the French physician and physiologist Étienne Jules Marey invented the photographic gun and chronophotography, creating “videos” of various animals in motion, such as dogs, cats, horses, and sheep. This major technological advance for studying motion and locomotion decomposed movement into series of consecutive photographic pictures (Marey, 1873). Philippson (1905) divided the dog’s walking cycle into several phases based on joint angular excursions and their transitions from flexion to extension. In the late 1960s, Engberg and Lundberg combined, for the first time, kinematic and electromyographic data during unrestrained locomotion in cats, establishing relationships between muscle activity and changes in joint angles (Engberg and Lundberg, 1969).

The study of locomotion requires characterizing kinematics, including variables, such as joint angles, the distances traveled by the limbs and temporal parameters, such as cycle and phase durations (Shen and Poppele, 1995; Nam et al., 2009). Video recordings capture these data by tracking limb movements using reflective markers placed on bony landmarks (Lavoie et al., 1995; Chau et al., 1998). Data are then analyzed by commercial or custom-made software to extract desired variables. The price of motion analysis systems varies but remains relatively high. Moreover, the use of software imposes a certain rigidity in the experience, as markers must be placed before the experiment. This makes it difficult to adjust to potential changes or to perform new measures unforeseen before recording. Placing markers on animals can be time-consuming and potentially disruptive to the animal (Bailey, 2018). Furthermore, errors in marker placement can arise due to movement of the skin over the joints during locomotion in animal models, especially in small rodents, but also larger models, such as the cat and dog (Goslow et al., 1973; Bauman and Chang, 2010). This can lead to inaccurate joint angle measurements, particularly at the knee. Finally, the analysis is subjected to human error. The performance of individual experimenters can vary within and between analysis sessions because of the repetitive nature of the task and the physical and mental fatigue caused by it (Weber et al., 1980). Variations in tagging accuracy between experimenters may also exist. There is thus a need to develop low-cost, flexible, and accurate motion analysis systems to characterize animal locomotion.

Since the 2000s, deep learning approaches, under the impetus of big tech companies, have been implemented in many areas. This technique, derived from machine learning, uses algorithms organized in several layers that allow the computer to learn by itself. Performance of the machine depends on the initial data and the accumulation of different experiences. Deep learning allows the creation of machines capable of beating the world go champion, a traditional Chinese strategy game (Silver et al., 2016), to recognize traffic signs on autonomous cars (Cireşan et al., 2012), and is applied in several scientific fields [see for review (Alipanahi et al., 2015; Hu et al., 2018)]. Motion and behavioral analyses have also seen the development of deep learning tools (Mathis and Mathis, 2020). Developed and made available in 2018 by a German-American research team, DeepLabCut^TM^ (DLC) was quickly adopted by many laboratories around the world as a reference tool to characterize behavior. DLC is an open-source software package using deep learning for motion tracking and markerless pose estimation. DLC is a deep convolutional network based on two key elements for pose estimation: deconvolutional layers and pretrained residual network (ResNet). It uses feature detectors of one of the best algorithms for human pose-estimation: DeeperCut (Mathis et al., 2018; Nath et al., 2019). With DLC, the user defines points of interest (e.g., specific body parts), which are followed throughout video recordings, allowing high-throughput locomotor screenings and frame-by-frame predictions. Thanks to its considerable flexibility, DLC can be adapted to a wide variety of models and studies, such as detailing cuttlefish predation techniques (Wu et al., 2020), evaluating manual dexterity during food-handling in mice (Barrett et al., 2020), or measuring bradykinesia in people with Parkinson’s disease (Williams et al., 2020). Implementing DLC in the laboratory is relatively cheap and fast. The software is open source, hence free of charge, and videos for analysis can be recorded from any device, independent of quality and without requiring advanced programming skills.

Recently, a research team developed a C#-based analysis tool coupled to DLC, called Visual Gait Lab (VGL). They tested this tool for gait analysis in the mouse model and compared their results to those commonly found in the literature (Fiker et al., 2020). VGL consists of two parts. The first one is a graphical user interface to install DLC and make it more user-friendly. Secondly, VGL comes with a tool to track paw placements. The software then integrates these results to measure certain gait variables, such as stride length and stance duration. The results calculated by VGL from estimates made on DLC were comparable to those in the literature. The DLC estimates were therefore valid for a mouse model. Is DLC for gait analysis also applicable to larger animal models and for other types of measures, such as joint angles?

The objective of the present study was to validate the accuracy of DLC for gait analysis in the adult cat by comparing results obtained with DLC and a custom-made software commonly used in cat studies [e.g., (Lavoie et al., 1995; Chau et al., 1998; Bouyer and Rossignol, 2003; Barriere et al., 2008; Martinez et al., 2013; Escalona et al., 2017)]. We also provide an Excel spreadsheet and MATLAB script for data analysis as Supplementary Material.

## Materials and Methods

### Animals and Ethical Information

The Animal Care Committee of the Université de Sherbrooke approved all procedures in accordance with the policies and directives of the Canadian Council on Animal Care (Protocol 442-18). In the present study, we used four intact adult cats (>1 year of age at the time of experimentation), two females and two males, weighing between 3.6 and 4.7 kg. We followed the ARRIVE guidelines for animal studies (Percie du Sert et al., 2020). To reduce the number of animals used in research, we used the cats in other studies to answer different scientific questions (Harnie et al., 2018, 2019, 2021; Merlet et al., 2020, 2021).

### Experimental Protocol

**FIGURE 1:**
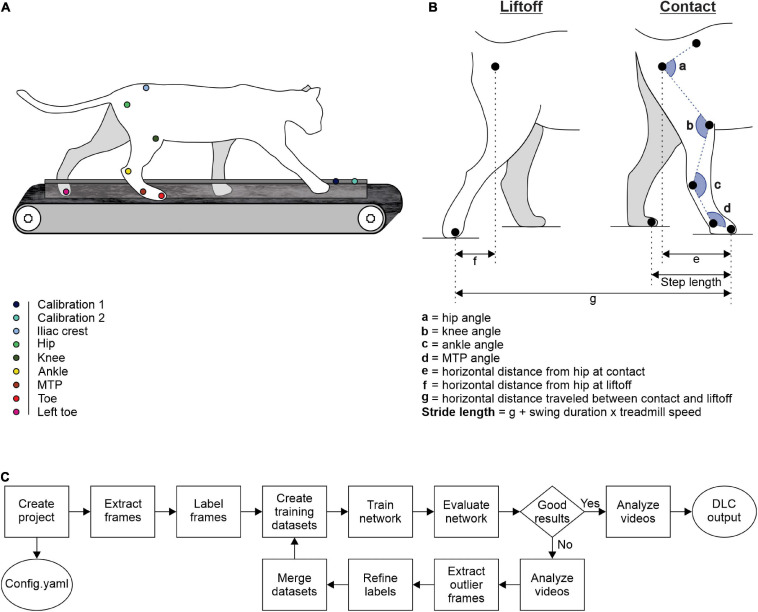
Experimental design and measured variables. **(A)** Reflective markers were placed on bony landmarks. The cat was then placed on a split-belt treadmill for video recordings during locomotion. **(B)** Lengths, distances, and angles measured at contact and at liftoff. **(C)** Schematic diagram summarizing DLC steps (adapted from Nath et al., 2019).

We trained cats to step on an animal treadmill composed of two independently controlled running surfaces 120 cm long and 30 cm wide (Bertec Corporation, Columbus, OH, United States) using positive reinforcement (food, affection) (Figure 1A). A Plexiglas separator (120 cm long, 3 cm high, and 0.5 cm wide) placed between the left and right belts ensured that the left and right limbs stepped on separate belts. Cats performed two types of quadrupedal locomotion: (1) tied-belt locomotion from 0.4 to 1.0 m.s^–1^ in 0.1 m.s^–1^ increments with both belts moving at the same speed and (2) split-belt locomotion with the slow side stepping at 0.4 m.s^–1^ and the fast side stepping from 0.5 to 1.0 m.s^–1^ in 0.1 m.s^–1^ increments. We aimed to collect ∼15 cycles per trial. Reflective markers were placed on the skin over bony landmarks: the iliac crest, greater trochanter, lateral malleolus, metatarsophalangeal joint (MTP), and at the tip of the toe.

### Data Acquisition and General Analysis

Two cameras (Basler AcA640-100g) captured videos of the left and right sides at 60 frames per second with a spatial resolution of 640 × 480 pixels. We analyzed each video off-line using our custom-made software called Expresso and with DLC. We determined contact and liftoff of the right hindlimb by visual inspection. We defined contact as the first frame where the paw made visible contact with the treadmill surface while liftoff corresponded to the frame with the most caudal displacement of the toe. We analyzed several spatial parameters, such as the distance between the toe and hip at contact and at liftoff, step length, and stride length (Figure 1B). As the cat does not travel actual distances on the treadmill, stride lengths were defined as the distance between contact and liftoff of the limb added to the distance traveled by the treadmill during the swing phase. It was calculated by multiplying swing duration by treadmill speed (Courtine et al., 2005; Thibaudier and Frigon, 2014; Dambreville et al., 2015). We also measured joint angles for the hip, knee, ankle, and MTP at contact and liftoff (Figure 1B). We analyzed each variable from 2,500 cycles of quadrupedal locomotion in the four intact cats.

### Custom Software

The custom software, Expresso, was developed in the laboratories of Trevor Drew and Serge Rossignol for gait analysis in the cat. It tracks reflective markers positioned on a limb. Here, we used the right hindlimb. At first, the experimenter specified the position of the reflective markers on 3–5 frames at the beginning of the video. The software then tags each reflective marker for all following frames. Expresso extrapolates knee position from the reflective markers of the greater trochanter and lateral malleolus. The positioning of the tags is often inaccurate for several frames, and we must reposition them manually frame-by-frame. The calculation of spatial values, such as step length, stride length, and the distance between the toe and hip requires manual tagging, in each desired frame, of the markers or regions of interest. For calibration, we used two markers placed in the same plane as the cat spaced 10 cm apart.

After properly placing all tags, the software exports several files. A first file provides values of angles. In addition, the software provides a graph showing the evolution of each angle during a step cycle, averaged over all cycles whose values are interpolated over 256 bins. Another file contains X and Y coordinates in cm of the manually noted points (i.e., the greater trochanter reflective marker and the tip of the right and left toes). This allows measurement of variables, such as the distance between the toe and hip at contact and liftoff, as well as step and stride lengths in Excel or MATLAB. Expresso also exports a file that lists all the frames determined by visual inspection where contacts and liftoffs of the right hindlimb occurred.

### DeepLabCut^TM^ (DLC)

We installed DLC on a computer running a Windows OS with Intel Core i7 8700 3.2 GHz, 12Mo (Intel^®^) processor and a GeForce RTX 2070 8 GB graphics card (Asus^®^) with a graphics processing unit (GPU) by Nvidia^®^. We then installed the latest driver for our graphics card, the Nvidia CUDA package allowing graphical computations and TensorFlow, an open-source software library created by Google for machine learning and deep learning. We implemented DLC 2.1.8.2. according to the development team’s recommendations (Nath et al., 2019) in an Anaconda environment, called DLC-GPU with its specifications available online^1^. DLC is written in Python 3.6.x.

The training with DLC consisted of several steps. First, we created our project defined by a project name, a username and a set of videos to create a training dataset. A config.yaml file is created, allowing us to define our points of interest or “body parts,” as DLC calls them, or to set the number of frames to extract. DLC then extracted 30 random frames per video from a total of 30 videos. Videos from all four animals during tied- and split-belt locomotion were used, including at least one video at each speed. We determined the number of frames to be extracted per video *a priori* with pilot testing. Via the DLC interface, we marked on each selected frame, the location of our points of interest. DLC tracked six points of interest on the right hindlimb, one on the left hindlimb and two calibration points, spaced 10 cm apart, allowing pixel-to-cm conversion. All of the labeled frames were available for visual inspection and could be corrected if needed. The set of labeled frames provided a training dataset and served as a basis for a pre-trained network (ResNet). The pre-trained network was refined end-to-end to adapt its weights to predict desired features, such as our points of interest. Then the network trained with over 1,030,000 iterations, generally taking between 8 and 12 h where DLC ran autonomously (e.g., overnight). We then evaluated the performance of the trained network on the training and test frames. The trained network analyzed videos, generating extracted pose files. The software allows several outputs, including a video with points of interest on each frame that we can link to form a skeleton. In addition to this visual output, DLC exports an excel file containing, for each video frame, X and Y coordinates of each point of interest in pixels as well as the likelihood (from 0 to 1) that the point of interest was properly positioned, in accordance with the training dataset and the other video frames. We then moved on to the network refinement stage. If the trained network did not appropriately generalize to unseen data in the evaluation and analysis step, we extracted additional frames with inadequate results and predicted labels were manually corrected. DLC extracted, from each video, 30 random frames among the putative outlier frames. We then replaced the misplaced points on the selected frames to create an additional set of annotated images that could be merged with the original dataset. This active-learning loop can be done iteratively to robustly and accurately analyze videos with potentially large variability (i.e., experiments that include many individuals over long time periods). The network was then trained again with the new updated dataset with the same number of iterations. Once the training was deemed optimal, we analyzed the other remaining videos with this training. The set of code lines required for these operations is available by the DLC development team (Nath et al., 2019). A quick schematic summary of the DLC methodology is shown in Figure 1C.

### Measurements of Gait Variables Using DLC Outputs

To obtain gait variables, we integrated DLC outputs containing coordinates of points of interest into a custom-made Excel program. This template is available as Supplementary Material. With this program, we can calculate gait variables of interest from the coordinates exported by DLC. The lengths, first expressed in pixels, are converted into cm using the ratio calculated from calibration markers (Figure 1B). The lengths in cm are then used to calculate joint angles. We can also calculate the speed of movement during overground locomotion or measure movement variables, such as stride length, on a treadmill by indicating the speed of the treadmill. Finally, to obtain average curves showing the evolution of joint angles over a cycle, the values of angles calculated frame-by-frame by the program are interpolated on 256 bins using a short MATLAB script. The Excel spreadsheet and MATLAB script are available as Supplementary Material. The last spreadsheet of the Excel file contains instructions for use.

### Statistical Analysis

We tested gait variables for normality using quantile-quantile plots (QQ-plots), which is a visual method to qualitatively assess the normality of the distribution of a data set. The abscissa of each point is equal to its value and the ordinate to the value that would correspond to this point if the distribution of the data was completely normal and followed a perfect Gaussian. If the center of the scatter plot formed by the set of points is on the line *y* = *x* (dotted red line in our graphs), with a slight deviation only present at the extremities, we can assume a normal distribution. In contrast, a large deviation at either end indicates significant influence of extreme values on the data’s distribution, making it non-normal (Wilk and Gnanadesikan, 1968; Pleil, 2016).

When finding a normal distribution, we compared the results of Expresso and DLC using two-way mixed effects, absolute agreement and single rater intraclass correlation coefficient (ICC). Several scoring systems have been used to categorize ICC estimates (Cicchetti, 1994; Koo and Li, 2016). We used the one proposed by Koo and Li (2016), which is stricter and with higher standards to assess agreement. This scoring system defines four levels of agreement according to the ICC score: less than 0.5 represents poor agreement, between 0.5 and 0.75 is moderate, between 0.75 and 0.9 is good and if the score exceeds 0.9, the agreement is considered excellent. As the ICC is only valid for normally distributed data, we used a non-parametric equivalent for data not normally distributed, termed Lin’s concordance correlation coefficient (CCC) (Lin, 1989, 2000). As the CCC is different from the ICC, we used another scoring system. For this test, a score of less than 0.90 represents poor agreement, between 0.90 and 0.95 is moderate, between 0.95 and 0.99 is substantial and an almost perfect agreement corresponds to values over 0.99 (McBride, 2005). We also measured Pearson’s correlation coefficient (CC) between the values obtained with Expresso and DLC to assess the linear relationship between the two measures. This statistical test is possible for both normally and non-normally distributed data. A conventional interpretation of the CC is: a score between 0 and 0.10 represents a negligible correlation, between 0.10 and 0.39 is a weak correlation, between 0.40 and 0.69 is a moderate correlation, between 0.70 and 0.89 is a strong correlation, and between 0.90 and 1 is a very strong correlation (Schober et al., 2018).

Finally, we also assessed the agreement between Expresso and DLC for each parameter using Bland–Altman plots with means ± standard deviations. The abscissa of each point is the mean value between its estimates by Expresso and DLC and the ordinate is the difference between the estimate by Expresso and DLC. The mean of these differences as well as the interval ±1.96 SD are also represented to visualize the agreement between the two methods according to the user’s conditions, and to detect visual bias or an anomaly in our agreement measurements. For example, if most points occur above or below the axis *y* = 0, it indicates a computational bias (over or underestimation) in one of the two methods. In contrast, a homogeneous distribution of data around the line *y* = 0 shows consistency between measures obtained with the two methods and the agreement measured (Giavarina, 2015; Kalra, 2017). Analyses were made with SPSS Statistics 20.0 (IBM Corp., Armonk, NY, United States).

## Results

The results reported here are based on an analysis of 2,500 cycles obtained from 177 trials in four adult cats (41–47 trials per cat). We pooled data from tied-belt and split-belt locomotion at different speeds and slow-fast speed differences, respectively.

### Step Length/Stride Length

**FIGURE 2:**
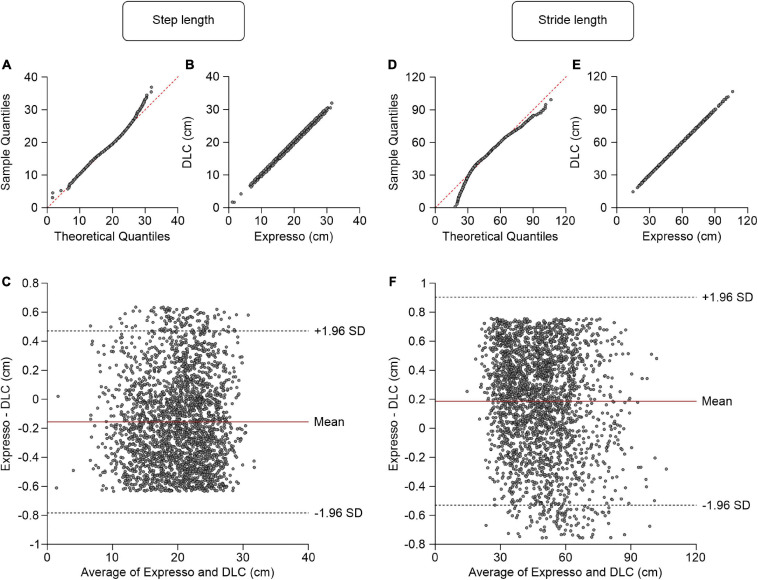
Comparison of step and stride lengths measured with data from Expresso and DLC. **(A)** Step length QQ-plot where the red dashed line represents a perfect normal distribution. **(B)** Step length scatter plot of values obtained with Expresso and DLC. **(C)** Bland–Altman plot of step length measurements from Expresso and DLC. The solid red line is the mean difference between the two methods and black dashed lines show ±1.96 standard deviations. **(D)** Stride length QQ-plot where the red dashed line represents a perfect normal distribution. **(E)** Stride length scatter plot of values obtained with Expresso and DLC. **(F)** Bland–Altman plot of stride length measurements from Expresso and DLC. The solid red line is the mean difference between two methods and black dashed lines show ±1.96 standard deviations.

We defined step length as the distance between the left and right hindpaws at right hindlimb contact. Average step length values measured by Expresso and DLC were 19.84 cm and 19.99 cm, respectively (Table 1). The QQ-plot follows the *y* = *x* line, showing only a slight deviation at its upper end (Figure 2A). We assumed a normal distribution for these data. The ICC score of 0.997 indicates excellent agreement, with a CC of 0.998 (Table 1 and Figure 2B). The homogeneous distribution of points around the *y* = 0 line on the Bland–Altman plot shows no bias between Expresso and DLC measurements (Figure 2C).

**TABLE 1:** Summary of Expresso and DLC comparisons.

Variables	Expresso	DLC	ICC	CC
Step length (cm)	19.84 ± 4.78	19.99 ± 4.77	0.997 [0.995–0.998]	0.998*
Hip distance at contact (cm)	11.55 ± 1.58	11.48 ± 1.59	0.987 [0.984–0.990]	0.988*
Hip distance at liftoff (cm)	−14.39 ± 3.81	−14.33 ± 3.80	0.998 [0.998–0.998]	0.998*
Hip angle at liftoff (cm)	135.21 ± 13.67	135.69 ± 13.60	0.971 [0.968–0.973]	0.971*
Knee angle at contact (°)	114.97 ± 6.39	115.20 ± 5.86	0.870 [0.860–0.880]	0.874*
Knee angle at liftoff (°)	118.43 ± 11.51	121.02 ± 11.02	0.891 [0.786–0.934]	0.915*
Ankle angle at contact (°)	115.14 ± 10.09	112.85 ± 9.21	0.919 [0.766–0.960]	0.948*
Ankle angle at liftoff (°)	139.30 ± 12.82	139.62 ± 12.29	0.973 [0.971–0.975]	0.974*
MTP angle at contact (°)	154.12 ± 8.05	154.11 ± 7.31	0.902 [0.894–0.909]	0.906*
MTP angle at liftoff (°)	155.03 ± 9.37	156.10 ± 9.80	0.879 [0.860–0.895]	0.885*

**Variables**	**Expresso**	**DLC**	**CCC**	**CC**

Stride length (cm)	48.23 ± 14.43	48.04 ± 14.49	1.000 [0.999–1.000]	1.000*
Hip angle at contact (°)	92.19 ± 11.50	94.17 ± 10.81	0.937 [0.933–0.941]	0.954*

For Expresso and DLC, data are presented as the mean ± standard deviation. The table indicates values of the intraclass correlation coefficient (ICC), the concordance correlation coefficient (CCC), and Pearson’s correlation coefficient (CC). *Significant at level 0.01.

Average stride length values measured by Expresso and DLC were 48.23 cm and 48.04 cm, respectively (Table 1). The QQ-plot shows a small deviation at its upper end and a larger one at its lower end (Figure 2D). We cannot assume a normal distribution for these data. We therefore measured the agreement using the CCC. The CCC score of 1.000 indicates very strong agreement with a CC also of 1.000 (Table 1 and Figure 2E). The homogeneous distribution of points around the *y* = 0 line on the Bland–Altman plot shows no bias between Expresso and DLC measurements (Figure 2F).

### Distance Between the Toe and Hip at Contact and Liftoff

**FIGURE 3:**
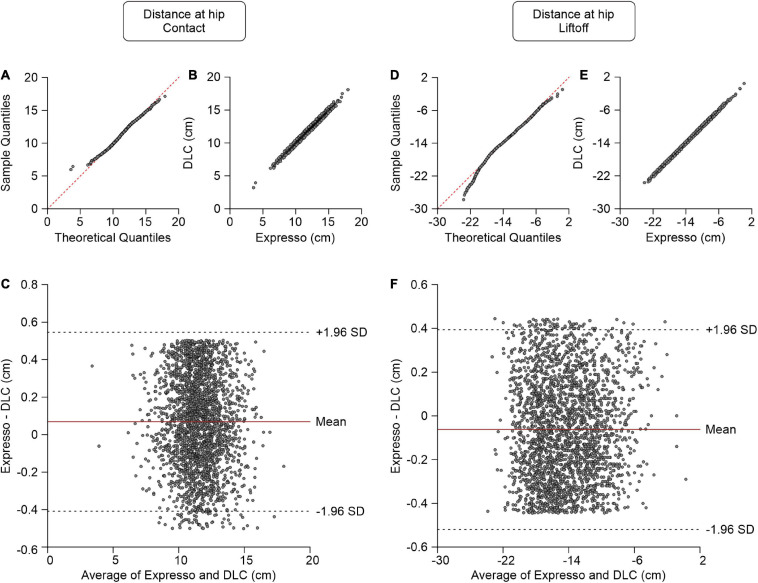
Comparison of distance at hip at contact and at liftoff measured with data from Expresso and DLC. **(A)** Distance at hip at contact QQ-plot where the red dashed line represents a perfect normal distribution. **(B)** Distance at hip at contact scatter plot of values obtained with Expresso and DLC. **(C)** Bland–Altman plot of distance at hip at contact measurements from Expresso and DLC. The solid red line is the mean difference between the two methods and black dashed lines show ±1.96 standard deviations. **(D)** Distance at hip at liftoff QQ-plot where the red dashed line represents a perfect normal distribution. **(E)** Distance at hip at liftoff scatter plot of values obtained with Expresso and DLC. **(F)** Bland–Altman plot of distance at hip at liftoff measurements from Expresso and DLC. The solid red line is the mean difference between two methods and black dashed lines show ±1.96 standard deviations.

The distance between the toe and hip is the horizontal distance between the right hindpaw toe marker and the downward projection of the hip marker. At contact, the paw is rostral to the hip and the distance value is positive, while at liftoff it is generally negative (i.e., caudal to the hip marker). Values of the average distance between the toe and hip at contact measured by Expresso and DLC were 11.55 cm and 11.48 cm, respectively (Table 1). The QQ-plot follows the *y* = *x* line in its center and shows no apparent deviation (Figure 3A), consistent with a normal distribution. The ICC score of 0.987 indicates excellent agreement, with a CC of 0.988 (Table 1 and Figure 3B). The homogeneous distribution of points around the *y* = 0 line on the Bland–Altman plot shows no bias between Expresso and DLC measurements (Figure 3C).

The values of the average distance between the toe and hip at liftoff measured by Expresso and DLC were −14.39 cm and −14.33 cm, respectively (Table 1). The QQ-plot follows the *y* = *x* line, showing only a slight deviation at its lower end (Figure 3D). We assumed a normal distribution for these data. The ICC score of 0.998 indicates excellent agreement, with a CC of 0.998 (Table 1 and Figure 3E). The homogeneous distribution of points around the *y* = 0 line on the Bland–Altman plot shows no bias between Expresso and DLC measurements (Figure 3F).

### Joint Angles

**FIGURE 4:**
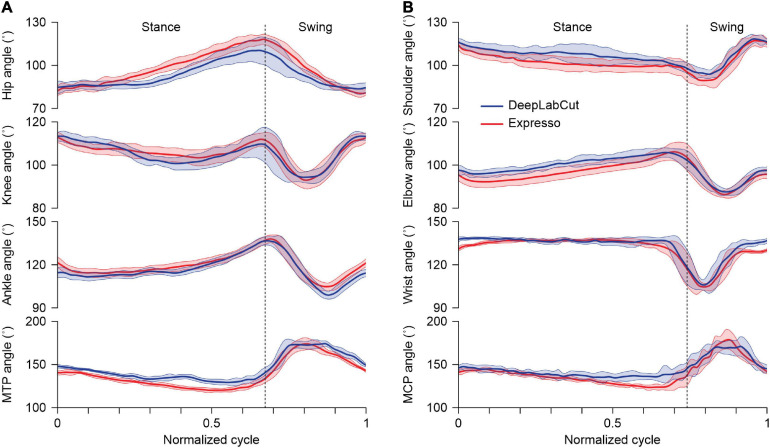
Averaged angular displacements for the right hindlimb and forelimb across the step cycle. **(A)** Averaged angular displacements (mean ± SD) at the hip, knee, ankle, and metatarsophalangeal (MTP) joints in the right hindlimb of an intact adult cat at a tied-belt speed of 0.4 m/s (*n* = 14 cycles). **(B)** Averaged angular displacements (mean ± SD) at the shoulder, elbow, wrist, and metacarpophalangeal (MCP) joints in the right forelimb of an intact adult cat at a tied-belt speed of 0.4 m/s (*n* = 14 cycles). The vertical dotted lines mark swing onset in **(A,B)**.

With Expresso and DLC, we calculated joint angle values on a frame-by-frame basis to determine their evolution over the step cycle. For the hip, knee and ankle, a decreasing angle indicates flexion (hip and knee) or dorsiflexion (ankle and MTP) of the joint. Figure 4A shows an example of hindlimb joint angles from one cat stepping at a treadmill speed of 0.4 m.s^–1^. Hindlimb joint angles over the step cycle were similar with both methods despite slight differences of a few degrees in certain parts of the cycle. To illustrate that DLC provides similar data as Expresso for the forelimbs, we measured joint angles at the shoulder, elbow, wrist, and metacarpophalangeal joints. As can be seen, both approaches generated similar forelimb joint angles across the step cycle (Figure 4B). We did not perform additional statistical comparisons for forelimb joint angles.

### Hip Angle

**FIGURE 5:**
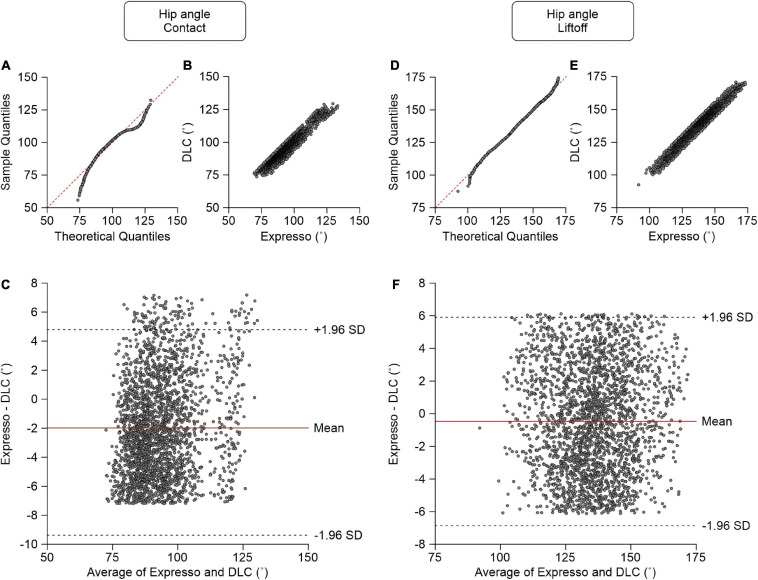
Comparison of hip joint angles at contact and at liftoff measured with data from Expresso and DLC. **(A)** Hip angle at contact QQ-plot where the red dashed line represents a perfect normal distribution. **(B)** Hip angle at contact scatter plot of values obtained with Expresso and DLC. **(C)** Bland–Altman plot of hip angle at contact measurements from Expresso and DLC. The solid red line is the mean difference between the two methods and black dashed lines show ±1.96 standard deviations. **(D)** Hip angle at liftoff QQ-plot where the red dashed line represents a perfect normal distribution. **(E)** Hip angle at liftoff scatter plot of values obtained with Expresso and DLC. **(F)** Bland–Altman plot of hip angle at liftoff measurements from Expresso and DLC. The solid red line is the mean difference between two methods and black dashed lines show ±1.96 standard deviations.

We measured the hip angle at contact and liftoff. At contact, average hip angle values measured by Expresso and DLC were 92.19° and 94.17°, respectively (Table 1). The QQ-plot shows two significant deviations at both ends (Figure 5A), and thus we cannot assume a normal distribution for these data. We therefore measured agreement using the CCC. The CCC score of 0.937 indicates moderate agreement, with a CC of 0.954 (Table 1 and Figure 5B). The homogeneous distribution of points around the *y* = 0 line on the Bland–Altman plot shows no bias between Expresso and DLC measurements (Figure 5C).

At liftoff, average hip angle values measured by Expresso and DLC were 135.21° and 135.69°, respectively (Table 1). The QQ-plot follows the *y* = *x* line at its center, with slight deviations at its extremes (Figure 5D). We assumed a normal distribution for these data. The ICC score of 0.971 indicates excellent agreement, with a CC of 0.971 (Table 1 and Figure 5E). The homogeneous distribution of points around the *y* = 0 line on the Bland–Altman plot shows no bias between Expresso and DLC measurements (Figure 5F).

### Knee Angle

**FIGURE 6:**
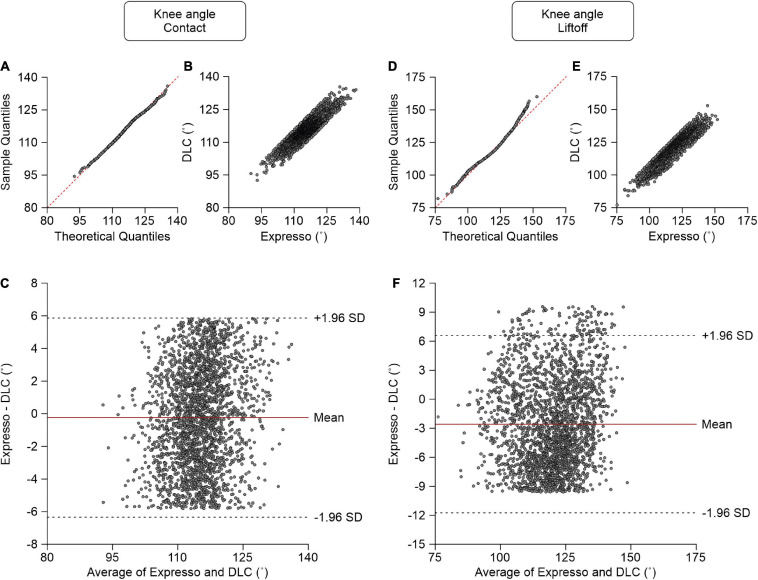
Comparison of knee joint angles at contact and at liftoff measured with data from Expresso and DLC. **(A)** Knee angle at contact QQ-plot where the red dashed line represents a perfect normal distribution. **(B)** Knee angle at contact scatter plot of values obtained with Expresso and DLC. **(C)** Bland–Altman plot of knee angle at contact measurements from Expresso and DLC. The solid red line is the mean difference between the two methods and black dashed lines show ±1.96 standard deviations. **(D)** Knee angle at liftoff QQ-plot where the red dashed line represents a perfect normal distribution. **(E)** Knee angle at liftoff scatter plot of values obtained with Expresso and DLC. **(F)** Bland–Altman plot of knee angle at liftoff measurements from Expresso and DLC. The solid red line is the mean difference between two methods and black dashed lines show ±1.96 standard deviations.

At contact, average knee angle values measured by Expresso and DLC were 114.97° and 115.20°, respectively (Table 1). The QQ-plot shows no major deviation and its center follows almost perfectly the *y* = *x* line (Figure 6A), consistent with a normal distribution. The ICC score of 0.870 indicates good agreement, with a CC of 0.874 (Table 1 and Figure 6B). The homogeneous distribution of points around the line *y* = 0 line on the Bland–Altman plot shows no bias between Expresso and DLC measurements (Figure 6C).

At liftoff, average hip angle values measured by Expresso and DLC were 118.43° and 121.02°, respectively (Table 1). The QQ-plot follows the *y* = *x* line at its center and shows a slight deviation at its upper end (Figure 6D). We can assume a normal distribution for these data. The ICC score of 0.891 indicates good agreement, with a CC of 0.915 (Table 1 and Figure 6E). The homogeneous distribution of points around the *y* = 0 line on the Bland–Altman plot shows no bias between Expresso and DLC measurements (Figure 6F).

### Ankle Angle

**FIGURE 7:**
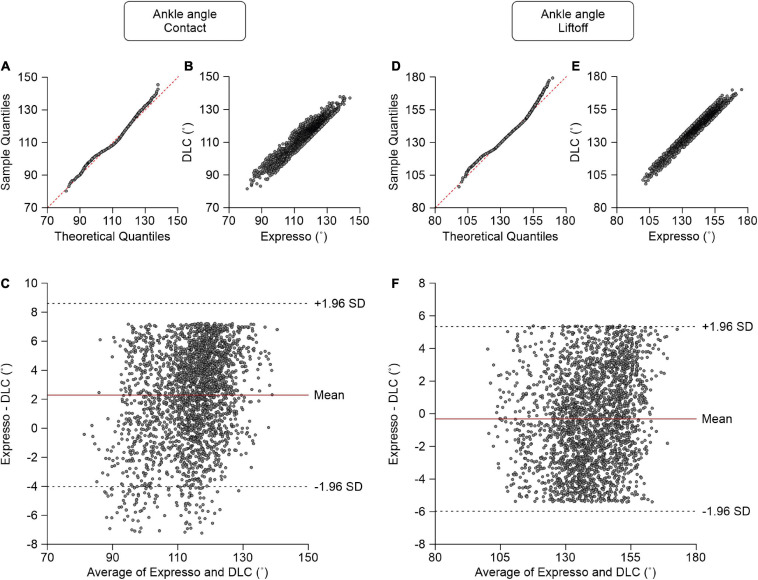
Comparison of ankle joint angles at contact and at liftoff measured with data from Expresso and DLC. **(A)** Ankle angle at contact QQ-plot where the red dashed line represents a perfect normal distribution. **(B)** Ankle angle at contact scatter plot of values obtained with Expresso and DLC. **(C)** Bland–Altman plot of ankle angle at contact measurements from Expresso and DLC. The solid red line is the mean difference between the two methods and black dashed lines show ±1.96 standard deviations. **(D)** Ankle angle at liftoff QQ-plot where the red dashed line represents a perfect normal distribution. **(E)** Ankle angle at liftoff scatter plot of values obtained with Expresso and DLC. **(F)** Bland–Altman plot of ankle angle at liftoff measurements from Expresso and DLC. The solid red line is the mean difference between two methods and black dashed lines show ±1.96 standard deviations.

At contact, average ankle angle values measured by Expresso and DLC were 115.14° and 112.85°, respectively (Table 1). The QQ-plot shows only slight deviations at its lower and upper ends and its center follows almost perfectly the *y* = *x* line (Figure 7A). We assumed a normal distribution for these data. The ICC score of 0.919 indicates excellent agreement, with a CC of 0.948 (Table 1 and Figure 7B). The relatively homogeneous distribution of points around the *y* = 0 line on the Bland–Altman plot does not seem to show any bias between Expresso and DLC measurements (Figure 7C).

At liftoff, average ankle angle values measured by Expresso and DLC were 139.30° and 139.62°, respectively (Table 1). The QQ-plot shows two slight deviations at both ends but its center follows the *y* = *x* line (Figure 7D). We can assume a normal distribution for these data. The ICC score of 0.973 indicates excellent agreement, with a CC of 0.974 (Table 1 and Figure 7E). The homogeneous distribution of points around the *y* = 0 line on the Bland–Altman plot shows no bias between Expresso and DLC measurements (Figure 7F).

### MTP Angle

**FIGURE 8:**
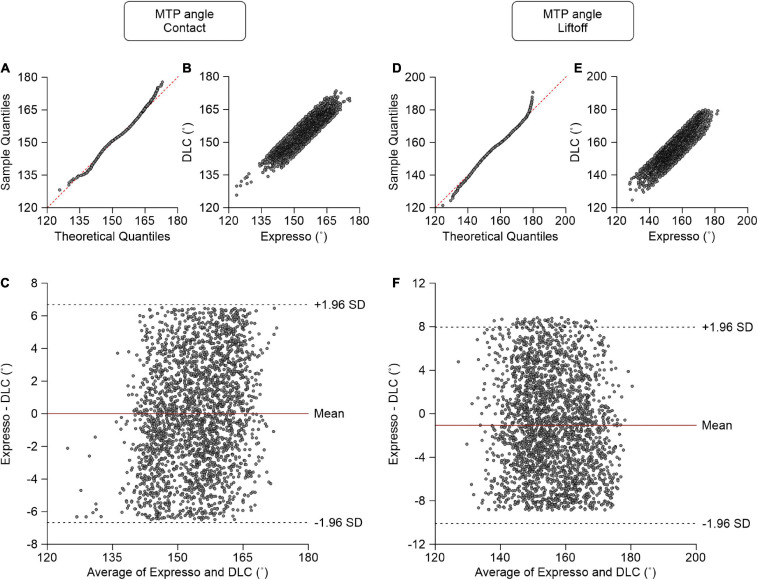
Comparison of metatarsophalangeal joint angles at contact and at liftoff measured with data from Expresso and DLC. **(A)** MTP angle at contact QQ-plot where the red dashed line represents a perfect normal distribution. **(B)** MTP angle at contact scatter plot of values obtained with Expresso and DLC. **(C)** Bland–Altman plot of MTP angle at contact measurements from Expresso and DLC. The solid red line is the mean difference between the two methods and black dashed lines show ±1.96 standard deviations. **(D)** MTP angle at liftoff QQ-plot where the red dashed line represents a perfect normal distribution. **(E)** MTP angle at liftoff scatter plot of values obtained with Expresso and DLC. **(F)** Bland–Altman plot of MTP angle at liftoff measurements from Expresso and DLC. The solid red line is the mean difference between two methods and black dashed lines show ±1.96 standard deviations.

At contact, average MTP angle values measured by Expresso and DLC were 154.12° and 154.11°, respectively (Table 1). The QQ-plot shows a slight deviation at its upper end but its center follows the *y* = *x* line (Figure 8A). We assumed a normal distribution for these data. The ICC score of 0.902 indicates excellent agreement, with a CC of 0.906 (Table 1 and Figure 8B). The homogeneous distribution of points around the *y* = 0 line on the Bland–Altman plot shows no bias between Expresso and DLC measurements (Figure 8C).

At liftoff, average MTP angle values measured by Expresso and DLC were 155.03° and 156.10°, respectively (Table 1). The QQ-plot has small deviations at its extremities and its center follows the *y* = *x* line (Figure 8D). We can assume normal distribution of these data. The ICC score of 0.879 indicates good agreement, with a CC of 0.885 (Table 1 and Figure 8E). The homogeneous distribution of points around the *y* = 0 line on the Bland–Altman plot shows no bias between Expresso and DLC measurements (Figure 8F).

## Discussion

The goal of the present study was to determine if measures of gait variables obtained with DLC agree or differ with those of a custom-made software that has been used in several cat studies. We focused on variables commonly used in gait analysis, including step and stride lengths, the horizontal distance of the toe from the hip at contact and liftoff, as well as hindlimb joint angles. Data are available upon request, including cat videos.

### Agreement Between Measures

In this study, we showed that the estimates provided by DLC agreed closely with those obtained with Expresso, a custom-made software. Indeed, nine of the 12 variables measured showed excellent agreement between DLC and Expresso based on ICC or CCC scores.

Three joint angle values measured at the knee and MTP joints did not meet excellent agreement but still qualified as good. The knee angle at contact (ICC score: 0.870) and at liftoff (ICC score: 0.891) qualified as good agreement. Even if the agreement scores were lower for these variables, they were still within an acceptable range. Indeed, it has been suggested that it is important that the lower limit of the 95% confidence interval of the ICC score is at least 0.75 (Burdock et al., 1963; Lee et al., 1989). In our study, the variables with excellent agreement and those with only good agreement fulfilled this condition (Table 1). Thus, despite somewhat lower agreement scores between Expresso and DLC for certain variables, they remained relatively high.

The lower agreement of knee joint angles could be because the skin moves around this joint during locomotion, changing its relative position (Goslow et al., 1973; Kuhtz-Buschbeck et al., 1994). Because the knee’s measured position with Expresso is extrapolated, it may be less accurate than training based on direct knee tags on a series of images with DLC. The knee joint remains easily visible on a well-shaved cat limb during locomotion.

The other joint where the agreement is not excellent is the MTP. Indeed, at liftoff, the agreement was good (ICC score: 0.879) and excellent at contact (ICC score: 0.902). This can be explained by the variability of the position of the reflective marker at the tip of the toe. Indeed, this marker can easily be displaced when it rubs against the treadmill. Also, the tip of the toe is a more subjective location than the anatomical landmarks for other reflective markers. The position may vary slightly from one experiment to another and between animals. Thus, the estimated position is likely more consistent with DLC because it does not rely on marker position.

When considering all variables, the two methods did not systematically over- or underestimate the results, as shown in Table 1 and in the Bland–Altman plots. Furthermore, the variability of the measures by DLC and Expresso was similar, as shown by similar standard deviations obtained with the two methods (Table 1). Although the standard deviation was high for the length variables (i.e., step/stride lengths and distance at hip at contact and liftoff) this is because we pooled results from a range of speeds and left-right speed differences. Step and stride lengths and the relative position of the paw at liftoff increases with speed in cats (Halbertsma, 1983; Frigon et al., 2017).

Figure 4 shows averaged values of the hindlimb and forelimb joint angles over a step cycle in one cat at a tied-belt speed of 0.4 m/s, calculated with Expresso and DLC. For the knee angle, we observed a slightly greater decrease (flexion) with DLC compared to Expresso in mid-stance. Although we did not perform a statistical analysis to determine significant differences between DLC and Expresso, DLC appears to better detect small angle changes during locomotion. This is also visible for the hip during stance. The forelimb joint angles measured with DLC and Expresso also followed similar trajectories across the step cycle, suggesting that DLC is equally effective for forelimb measures.

### Advantages and Limitations

One advantage of DLC is that it allows for a more flexible *post-hoc* analysis. Indeed, the developers defined it as “markerless pose estimation of user-defined body parts with deep learning” (Mathis et al., 2018), which removes the rigidity imposed by placing reflective markers before experimentation. Locating anatomical points of interest and placing markers means anticipating all the variables to be measured prior to experimentation. However, by analyzing videos with DLC, we can identify points of interest not prepared for at the outset, allowing for greater freedom in the use and exploitation of video recordings. Beyond this flexibility in the analysis, DLC and its markerless use has other important advantages for gait analysis, especially in animals like the cat. Some animals can be difficult and restless when placing reflective markers. This can induce variability in the positioning of the markers because of the cat’s movement, particularly at distal joints. Restraining the cat can be a source of stress that makes the upcoming experience more difficult (e.g., animal is less cooperative and more aggressive). This can affect the cat’s locomotion and bias the results (Amat et al., 2016). Another advantage is that marker visibility is not required. For example, after a spinal cord injury, one or both hindpaws may drag on the walking surface during the stance-to-swing transition and part or all of the swing (Bélanger et al., 1996; Drew et al., 2002; Hurteau et al., 2015). Consequently, the camera can lose sight of the toe marker and it can easily come off. We are currently measuring kinematics using DLC in spinal-transected cats. The tracking by DLC is equal or better than for intact cats (unpublished observations), most likely because the animal mainly remains in the same position on the treadmill as weight support is provided by an experimenter and the forelimb are stationary. Additionally, the main output provided by DLC is simple, namely a spreadsheet containing predicted x and y coordinates of each point of interest frame-by-frame that can be used by various software. We chose an Excel spreadsheet to calculate our variables of interest from these points for practical reasons but it remains the choice of the experimenter.

Although we validated the use of DLC during treadmill locomotion, it has been used for a wide variety of movements, such as jumping in the spider (Brandt et al., 2021), reaching movements in the rat (Parmiani et al., 2019), or cuttlefish predation techniques (Wu et al., 2020). Studies that focus on non-locomotor rhythmic movements would also benefit from DLC. For instance, the paw-shake, which consists of rapid oscillatory movements of the paw when the underside of the paw contacts an irritant (Smith et al., 1985; Carter and Smith, 1986). The rapid and vigorous movements during paw-shake facilitate the loss of markers. DLC has already been validated for three commonly used behavioral tests in mice [e.g., open field test, elevated plus maze, and forced swim test (Sturman et al., 2020)] and balance analysis in human (Vonstad et al., 2020) against existing commercial solutions. In humans, there are more sophisticated approaches, such as optoelectronic systems that track light emitted (active) or reflected (passive) by markers, which is then converted to electrical signals to determine position in space (reviewed in Prakash et al., 2018). However, such systems are more expensive and complicated to implement.

A limitation of our current approach is that we only reconstructed the movement in 2D. However, DLC can be used for 3D analysis. With two cameras, it is possible to reconstruct in three dimensions an entire side of the body and a full 3D reconstruction of the body is possible with six cameras (Nath et al., 2019). The main limitation of DLC is that it requires a GPU to produce consistent results quickly. Although possible with a central processing unit (CPU), analysis speed will be considerably slower [divided by 10–100 (Mathis and Warren, 2018)]. Analysis speed also depends on video dimensions. A higher image definition means more pixels and a slower DLC analysis (Mathis and Warren, 2018). To target as many experimental designs as possible, DLC does not rely on a predefined body model. Thus, if points of interest are hidden and not visible throughout the video, tracking these points will be more complex. However, as DLC analysis is frame-by-frame, if the point of interest appears on at least some frames, then it will be possible to detect on frames where it is hidden (Nath et al., 2019).

To conclude, the mean values of the variables of interest obtained with DLC matched with high agreement those obtained with a commonly used custom-made software for cat locomotor studies. The use of DLC reduces inter- and intra-individual variability in marker placement, thanks to deep learning, while being suitable for any style of movement or gait study regardless of whether or not markers were placed. The implementation of deep learning in laboratories worldwide for gait and motion analysis, and more specifically DLC, seems to be a logical direction because it is faster, more accurate and more repeatable than existing commercial solutions while remaining easily deployable, flexible to the needs of the experimenter and relatively low cost.

## Data Availability Statement

The raw data supporting the conclusions of this article will be made available by the authors, without undue reservation.

## Ethics Statement

The animal study was reviewed and approved by the Comité Facultaire de Protection des Animaux de la Faculté de Médecine et des Sciences de la Santé (CFPA-FMSS).

## Author Contributions

CL and AF conceived and designed the research and drafted the manuscript. JH and AF performed the experiments. JA analyzed the data. CL performed the statistical analysis. CL, JA, and AF interpreted the results. CL, JH, and AF prepared the figures. CL, JA, JH, and AF edited and revised the manuscript and approved the final version of the manuscript. All authors contributed to the article and approved the submitted version.

## Conflict of Interest

The authors declare that the research was conducted in the absence of any commercial or financial relationships that could be construed as a potential conflict of interest.

## Publisher’s Note

All claims expressed in this article are solely those of the authors and do not necessarily represent those of their affiliated organizations, or those of the publisher, the editors and the reviewers. Any product that may be evaluated in this article, or claim that may be made by its manufacturer, is not guaranteed or endorsed by the publisher.

## Footnotes

1
https://github.com/DeepLabCut/DeepLabCut/blob/master/conda-environments
