# Supplementary figures and images for: A Validation of Supervised Deep Learning for Gait Analysis in the Cat
Source: Front Neuroinform. 2021 Aug 19;15:712623. doi: 10.3389/fninf.2021.712623 (PMC8417424; doi:10.3389/fninf.2021.712623)

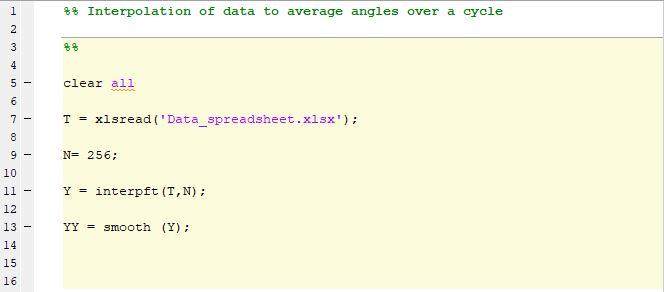

Supplement: Supplementary file 1 [file Image_1.jpeg]
